# Nanotechnology-Based Biopolymeric Oral Delivery Platforms for Advanced Cancer Treatment

**DOI:** 10.3390/cancers12020522

**Published:** 2020-02-24

**Authors:** Vanessa T. Chivere, Pierre P. D. Kondiah, Yahya E. Choonara, Viness Pillay

**Affiliations:** Wits Advanced Drug Delivery Platform Research Unit, Department of Pharmacy and Pharmacology, School of Therapeutic Sciences, University of Witwatersrand, Johannesburg, 7 York Road, Parktown 2193, South Africa; vanessachivere@gmail.com (V.T.C.); pierre.kondiah@wits.ac.za (P.P.D.K.); yahya.choonara@wits.ac.za (Y.E.C.)

**Keywords:** cancer, biopolymers, nanoparticles, targeted drug delivery, cancer nanotechnology, oral delivery

## Abstract

Routes of drug administration and their corresponding physiochemical characteristics play major roles in drug therapeutic efficiency and biological effects. Each route of delivery has favourable aspects and limitations. The oral route of delivery is the most convenient, widely accepted and safe route. However, the oral route of chemotherapeutics to date have displayed high gastric degradation, low aqueous solubility, poor formulation stability and minimum intestinal absorption. Thus, mainstream anti-cancer drugs in current formulations are not suitable as oral chemotherapeutic formulations. The use of biopolymers such as chitosan, gelatin, hyaluronic acid and polyglutamic acid, for the synthesis of oral delivery platforms, have potential to help overcome problems associated with oral delivery of chemotherapeutics. Biopolymers have favourable stimuli-responsive properties, and thus can be used to improve oral bioavailability of anti-cancer drugs. These biopolymeric formulations can protect gastric-sensitive drugs from pH degradation, target specific binding sites for targeted absorption and consequently control drug release. In this review, the use of various biopolymers as oral drug delivery systems for chemotherapeutics will be discussed.

## 1. Introduction

Majority of global deaths are caused by non-communicable diseases, with cancer ranking as the leading cause of death. In 2015, the World Health Organisation estimated that cancer is one of the leading causes of death in 91 of 172 countries [1]. Lung cancer is the most common type of cancer, with the highest number of new cases in 185 countries followed by breast, prostate and colon cancer, respectively [1]. Cancer-related deaths are projected to increase rapidly, with an estimated number of >20 million by the end of 2025 [2].

Current cancer treatment options include surgery, radiation therapy and/or chemotherapy, or a combination of these [3]. Conventional chemotherapy works by interfering with DNA synthesis in rapidly dividing cancer cells, leading to their death or impeded cell replication. Chemotherapy causes numerous side effects and is known to also affect healthy cells in the body [4]. The inevitable targeting of healthy cells is the major cause of the high mortality rate of cancer patients due to cellular toxicity. Another aggravating factor is the use of high doses of chemotherapeutic agents, since the bioavailability of several cancer drugs is relatively poor [4]. The use of high drug doses leads to elevated toxicity in normal cells, with the risk of drug resistance developing [4]. Most anti-cancer drugs are administered by intravenous infusion (IV) and, in majority of instances, require patients to be hospitalized leading to poor treatment compliance.

It was therefore necessary for researchers to explore the development of oral delivery platforms (ODPs) for cancer drugs in order to provide self-administered chemotherapy for the treatment of specific types of cancer. The oral route of drug administration is desirable because of its convenience, sustainability and superior patient compliance. However, numerous cancer drugs are not feasibly delivered via the oral route due to poor aqueous solubility, low oral bioavailability, gastric degradation (stability), the first-pass effect and high affinity binding to biological proteins.

Over the years, research has been undertaken on the use of various biopolymers as a framework for oral drug delivery. Ideally biopolymers should be non-immunogenic and improve the properties of anti-cancer drugs to be delivered via the oral route. At present, several biopolymer-based nanoparticle (NPs) have been developed as chemotherapeutics but unfortunately majority are for intravenous administration. For this reason, there is still a dire need for developing ODPs for cancer treatment.

The development of effective oral chemotherapeutics could revolutionise cancer therapy, as their use would provide control to the patient. Specialized biopolymers have been synthesized to design ODPs that can survive the harsh gastro-intestinal tract (GIT) conditions for improved anti-cancer drug stability and hence be able to effectively improve drug absorption and bioavailability of anti-cancer drugs [5]. ODPs for chemotherapy have the potential to reduce drug toxicity as doses can be adjusted (via dose banding) to maintain therapeutic concentrations for anti-cancer activity without elevating toxicity to healthy cells [5]. ODPs for chemotherapy have been developed using novel nanocarriers, such as, nanoliposomes, nanomicelles and nano-enabled hydrogels. The use of these nanocarriers ensures the protection of the anti-cancer drug from gastric degradation and boost bioavailability. Furthermore, surface modification of ODPs impart targeted delivery of the anti-cancer drugs [5]. The success of any future ODPs for chemotherapy requires both the use of biopolymer nanocarriers and an appropriate targeting mechanism to ensure effective bioavailability.

This review provides a concise incursion into the use of various biopolymers as ODPs in cancer therapeutics. It also provides an assimilation of the work undertaken in this area and highlights the gaps and challenges faced with the sustainable development of ODPs as a form of chemotherapy for specific cancers.

## 2. Biopolymers Used for the Development of ODPs in Cancer Treatment

Biopolymers have become the point of focus for anti-cancer drug delivery due to their biodegradability, biocompatibility and versatility that allows for controlled and targeted delivery for chemotherapeutics. Oral administration of chemotherapeutics is favoured in a myriad of cancer types, but it is limited by several challenges that mostly include drug degradation in the GIT, limited bioavailability, incomplete drug absorption and extensive hepatic metabolism for most anti-cancer drugs. Effective oral drug delivery for anti-cancer drugs is highly dependent on drug stability within the enzymatic and physicochemical GIT environment. As soon as the drug reaches the stomach, its stability is affected by the acidic environment and digestive enzymes are continuously secreted along the GIT that can degrade chemotherapeutic drugs before reaching target sites [6]. The unique properties of biopolymers may facilitate oral delivery of chemotherapeutics. For example, the bio-adhesive properties of some biopolymers make them exceptional candidates for promoting drug permeability across the mucosal membrane [6]. In addition, many have inherent properties that enable them to control the release of the incorporated drug using principals of improved solubility, stability and permeability, and reduced toxicity [6]. Table 1 provides a summary of the biopolymers used for the design of nano-enabled ODPs for anti-cancer drugs.

Polymers for ODP formulation include chitosan, gelatin, alginates, PLGA, poly(glutamic acid) (PGA) and hyaluronic acid. They have the capacity to achieve sustained drug release, which is of great interest, as it helps overcome tissue barriers until the drug reaches systemic circulation.

### 2.1. Protein-Based Biopolymers

Protein-based biopolymers are of great interest because they are water soluble, this makes them excellent candidates for use in the formulation of ODPs for hydrophobic chemotherapeutics [7]. ODPs using protein-based biopolymers are also essential in cancer therapy as they can enhance drug retention time, drug absorption and can be modified to impart targeted delivery [8]. Enhanced absorption and drug retention time lead to improve chemotherapeutic efficacy, while targeted delivery limits off-target cell toxicity. There are several examples of protein-based biopolymers with specific physicochemical properties for potential use in designing ODPs for anti-cancer drugs.

#### 2.1.1. Gelatin

**Figure 1:**
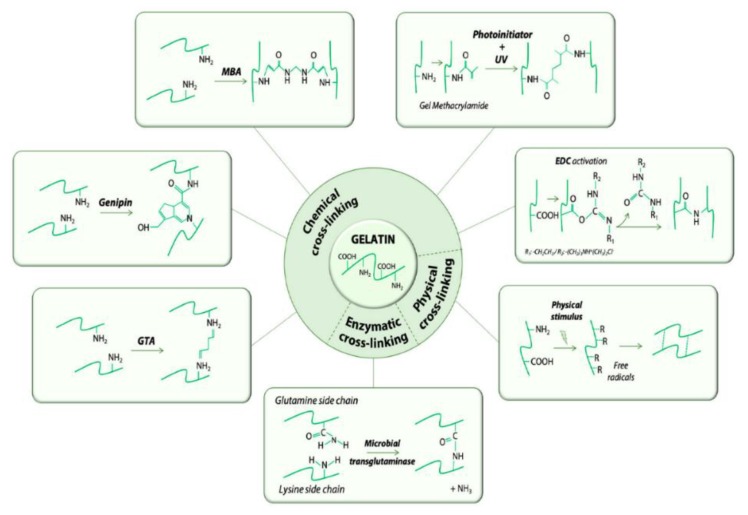
Schematic representation of crosslinking methods used to formulate gelatin nano-materials. Adapted and modified with permission from [14].

Gelatin is a natural biopolymer with favourable properties for delivery of chemotherapeutics. Gelatin is derived from collagen by acid/alkaline hydrolysis and has been used for the development of several nanocarriers as it is easy to crosslink with other compounds [9,10]. Gelatin has numerous ionisable groups and has notably resulted in the use of gelatin for colloidal drug delivery systems. Gelatin based platforms can be chemically crosslinked, as shown in Figure 1, by glutaraldehyde, genipin, carbodiimides and N-hydroxysuccinimide; making gelatin an exceptionally adaptable oral drug delivery biopolymer. Gelatin modification yields stealth carriers that can improve drug availability as the drug is shielded from degrading mechanisms, and is able to evade reticuloendothelial uptake, which is important for ODPs. Drugs can be protected from the acidic gastrointestinal tract conditions and enzymes, thus improving circulation time and accumulation in the leaky vasculature of tumour tissues [11]. Gelatin can also be modified to enhance drug encapsulation efficiency, which in turn, leads to improved bioavailability. Gelatin can be tailor modified to suit the drug being loaded and is therefore a good candidate for oral delivery of hydrophobic chemotherapeutics [11]. Another valuable advantage of gelatin modification is the incorporation of targeting moieties for targeted oral delivery; to spare healthy cells and ensure that after intestinal drug absorption, only the tumour cells are targeted and destroyed [12,13,14].

There has been a significant amount of research done into the use of gelatin for nano-formulations for the delivery of DNA molecules, cancer and tuberculosis agents.

Amit Singh et al. formulated EGFR-targeted redox-responsive gelatin NPs. The gelatin NPs were formulated for targeted delivery of gemcitabine (GEM), for the treatment of pancreatic cancer. The NPs were synthesised by ethanol induced solvation process, to encapsulate GEM. In order to enhance circulation time and enhance target specificity, the gelatin NPs were coated with poly(ethylene glycol) (PEG). The group carried out in vivo in vitro studies and the results obtained showed that the encapsulated GEM had a significantly improved cytotoxic profile against the cancer cells and the EFGR-targeted NPs could effectively orally deliver the GEM with improved efficacy as compared to an intravenous solution of GEM in succinimidyl 3-[2-pyridyldithio]-propionate) (SPDP). GEM-SPDP had an *IG*_50_ value of 8.39 ± 1.79 μM, as compared to the EGFR-targeted redox-responsive GEM loaded NPs, with an *IG*_50_ value of 17.08 ± 2.32 μM. The GEM-SPDP had a lower *IG*_50_ value but could not serve as a viable therapeutic option in vivo and in clinical use, as it was non-specific and displayed a high binding capacity to any protein in the body [15]. In another study carried out by Sajed Amjadi et al., gelatin was used for the formulation of PEGylated gelatin NPs for the co-delivery of doxorubicin (DOX) and betanin (BET). The aim was to enhance therapeutic efficacy of the chemotherapy, with reduced side effects.

The formulated NPs were pH-responsive and the loading capacity of DOX and BET was 20.5% and 16.25%, respectively. Shape and size of the NPs were analysed, and results showed that the formulated NPs were well rounded, with a uniform size around 162 nm. The pH-response profile was assessed in simulated physiological and tumour environments, and controlled release was shown by adjusting the environmental pH. The formulated NPs were further assessed for cell viability on breast cancer cells (MCF-7), in comparison to free-form DOX and BET. The results obtained showed a decrease in cell viability of the MCF-7 cells. Hence, the group concluded that the developed delivery platform is a promising nanocarrier for cancer treatment [16]. In a recent study, Uyen Vy Vo et al. formulated DOX loaded gelatin-poly(ethylene glycol) methyl ether (mPEG) functionalised porous nanosilica (PNS) NPs. The co-polymeric platform formulated was characterised and results showed that the NPs were spherical in shape, with an average size diameter of 69.60 ± 3.27 nm. The resultant DOX loaded NPs exhibited sustained release of DOX. At pH 7.4, 12.4% of DOX was released from the NPs in the first 2 h, and later there was a steady increase from 6 h (22.4%) to 36 h (32.3%). At a pH of 4.5, there was a drastic difference as approximately half of DOX was released from the formulated NPs within 6 h, and the release constantly increased until 36 h, at around 70%, thereafter remaining stable up to 96 h. These results led the group to conclude that DOX/gelatin-mPEG-PNS NPs have a high potential for effective oral delivery of DOX in cancer therapy [17]. Another group carried out a study on the loading efficiency of PNS NPs modified by gelatin, only. The group successfully formulated spherical DOX loaded PNS-gelatin NPs with an average diameter of 70 nm. They also assessed the drug loading efficiency (DLE) and drug release of the nanoparticles. The results showed a DLE of 55.90% and a sustained release profile at physiological pH 7.4. Therefore, they concluded that DOX/PNS-gelatin NPs were a promising delivery platform for pH-responsive release of DOX [18].

#### 2.1.2. Collagen

Collagen is a naturally occurring protein, abundantly present in animals. It has been used in various drug delivery system applications [19]. Collagen is a biopolymer of interest for use in oral drug delivery, as it is biodegradable, non-antigenic, non-toxic, and biocompatible and has shown synergism with other bioactive compounds. The functional groups in collagen can easily be modified to produce the desired oral drug delivery properties, and the biodegradability of the compound can be controlled by crosslinking. In addition, collagen has shown properties of being able to resemble the microenvironment of some tumours. This characteristic is beneficial in cancer therapy as it enables the collagen compound to be able to effectively infiltrate the tumour area, to deliver anti-cancer agents [20].

A study was carried out by Zhong Luo et al., where they formulated mesoporous silica NPs end capped with collagen. The aim of the study was to synthesise redox-responsive NPs for targeted drug delivery of anti-cancer drugs. Targeted delivery and cellular uptake studies were carried out on HepaG2 cells. The results obtained showed a 2.2-fold increase in targeted cellular uptake, as compared to the free drugs in intravenous solution. The group therefore concluded that collagen capped mesoporous silica NPs could successfully serve as redox-responsive nanocarriers. The formulated nanoparticles exhibited excellent biocompatibility, cell specific delivery and excellent cellular uptake properties. The collagen capped mesoporous silica NPs can potentially be used for oral chemotherapeutic targeted delivery [21].

### 2.2. Polyamino Acid-Based and Polyester-Based Biopolymers

#### 2.2.1. Polyglutamic Acid (PGA)

Polyglutamic acid (PGA) is a polyamino acid that can be used to form conjugates with anti-cancer drugs. These polymer–drug conjugates have the potential to increase chemotherapeutic drug efficacy and reduce toxicity towards normal cells. PGA is water soluble and is therefore a good candidate for orally delivering hydrophobic anti-cancer drugs. Additionally, PGA is non-toxic to both the environment and humans [22]. PGA is made up of repeating *D*-glutamic acid/*L*-glutamic acid units, and or a combination of both. The molecular weight of the polymer controls the release of the entrapped drug. Hence, polymers of varied molecular weights are needed, depending on the desired properties and applications of the polymer [23,24].

Attention has been drawn to γ-PGA because of its diverse properties. Desale et al. performed a study where they synthesised PEG-*b*-(PGA)-*b*-poly(phenylalanine) micelles for the dual delivery of paclitaxel (PTX) and an alkylating agent (CDDP). PTX was bound to the polyphenylalanine core, with 9% loading capacity while the CDDP was bound to the PGA layer, with 15% loading capacity. Therapeutic efficacy of the formulated co-delivery platform micelles was tested against A2780 ovarian cancer cells and results obtained showed improved efficacy, as compared to micelles containing PTX or CDDP alone. The group noted that besides multi-drug delivery capability of the PEG-*b*-(PGA)-*b*-poly(phenylalanine) micelles, the micelles also showed improved stability against disassembly in the gastric region and therefore enhanced tumour delivery [25]. In another study Yi-fei Li et al. evaluated the potential of cisplatin (CIS) loaded PGA-g-methoxy PEG nanoparticles. Analysis tests showed that the drug release rate was accelerated in acidic environment. In vitro results proved that the CIS/PGA-g-methoxy PEG NPs could inhibit proliferation of MNNG/Hos osteosarcoma cells. Results from in vivo analysis tests carried out in tumour-bearing mice, exhibited a comparably higher efficacy with lower toxicity effects as compared to free CIS in intravenous solution. Therefore, it was concluded that the CIS-NPs can be used as a potential osteosarcoma therapy [26].

In 2015, Liadong Deng et al. formulated chitosan-PGA-grafted PEG-DOX NPs. Their aim was to engineer drug gastric survivability, improve intestinal permeability, and enhance hemodynamic stability and intracellular activity of DOX. The formulated NPs demonstrated long-circulating properties, improved therapeutic efficacy and lower systemic toxicity as compared to DOX free in intravenous solution at a concentration of 10 μg/mL. The mucoadhesive properties of chitosan permit the formulated NPs to persist in the stomach and adhere to the mucus layer of the small intestine, and subsequently penetrate through the small intestine into the blood circulation. In vivo studies of the chitosan-PGA-grafted PEG-DOX NPs at a dose of 15 mg/kg, were carried out on Balb/c mice xenografted with Ehrlich ascites tumor, with DOX-HCL (15 mg/kg) intraperitoneal injection (i.p.) solution being used as the control formulation. The group treated with the oral NP formulation showed no deaths up to 4 weeks, while the group treated with i.p. solution had a survival rate of approximately 45.5% over the same period. The chitosan-PGA-grafted PEG-DOX NPs exhibited comparable tumour inhibition effects, as that of i.p. DOX-HCl, with significantly lower toxicity, enhanced cellular uptake and relative stability in physiological circulation. It was thus concluded that the chitosan-PGA-g-PEG/DOX NPs are a promising nano-platforms for oral delivery of chemotherapeutics [27].

#### 2.2.2. PLGA

Poly(lactic acid-co-glycolic acid) (PLGA) is a polyester, copolymer block made up of polyglycolic acid and poly-lactic acid [28]. It is a biocompatible, biodegradable and non-toxic polymer. Biodegradation of the copolymer means it can easily be removed from the body. Therefore, there is less risk of increasing toxicity while delivering anti-cancer drugs. PLGA nanoparticles are a promising platform for cancer therapy, with less side effects and improved drug efficacy [29].

Biodegradation of the copolymer occurs by bulk erosion. Its degradation mechanism allows for potential drug survivability in the acidic gastric environment, as the drug will be encapsulated at the core. The polymeric chains of PLGA are cleaved by hydrolysis and the monomeric units, lactic acid and glycolic acid, are metabolically excreted from the body [30]. The degradation products are non-toxic to living organisms.

**Figure 2:**
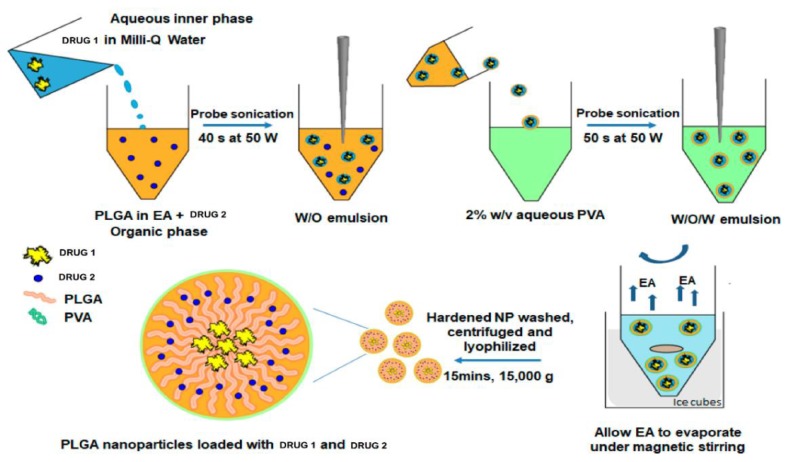
Example of a formulation method for the preparation of poly(lactic acid-co-glycolic acid) (PLGA) nanoparticles (NPs). Diagram shows preparation of PLGA NPs for the simultaneous delivery of two different drugs. The drug-loaded NPs are prepared by a double emulsion-solvent evaporation method. Abbreviations: PVA—polyvinyl alcohol; EA—ethyl acetate. Adapted and modified with permission from [31].

PLGA NPs can be formulated by use of emulsion evaporation, emulsion diffusion, solvent displacement and nanoprecipitation techniques. Figure 2 shows the use of a double emulsion-solvent evaporation technique in the formulation of a PLGA based co-delivery platform [31].

In 2017, Yan-na Cui et al. formulated paclitaxel (PTX)-loaded magnetic PLGA (50:50) NPs, surface modified with transferrin (TF). The aim of their research was to investigate the feasibility of the drug-loaded magnetic PLGA nanoparticles in enhancing cellular uptake and thus improving the therapeutic efficacy against cancer cells. The drug-loaded PLGA NPs were characterised in terms of encapsulation efficiency, size and morphology, magnetic properties, in vitro PTX release, and lastly, cytotoxicity and cellular uptake were evaluated in vitro against MCF-7 and U-87 cells. The morphology results obtained showed that the NPs were nearly spherical and approximately 150 ± 20 nm. Encapsulation efficiency (EE) was reported to be 82.9% and more than 80% of PTX was released over a period of 10 days, with the release profile following a biphasic profile. The formulated PTX-PLGA NPs showed increased cytotoxicity against the cancer cells and improved cellular uptake, as compared to the free PTX in solution for injection [32].

In a more recent study, Ajinder Kaushik et al. used PLGA 85:15 to formulate cetuximab (CMB) loaded PLGA NPs [33]. Their aim was to improve drug solubility, drug circulation time and enhance site specificity. The formulated NPs had a molecular size in the 115–270 nm range, PDI < 0.50. Biodistribution and pharmacokinetic studies were carried out in healthy strain mice. One group was intravenously injected with free labelled CMB in solution and another group was orally administered labelled formulated NPs. Various organs were harvested from both groups, 24 h post-administration. The results obtained demonstrated statistically significant findings between the two groups. Higher concentrations of CMB-loaded NPs were observed in the liver, blood, lungs, bone and brain tissues, as compared to that of the intravenously administered CMB. Oral administration of the CMB-loaded NPs may therefore be useful in treatment of tumours in these tissues. Lower distribution of the NPs to the heart and kidneys were reported, thus proving advantageous, in reducing side effects and toxicity. The results from the analytical tests carried out, led this team to conclude that the oral CMB-PLGA NPs showed sustained release and improved drug targeting, as compared to the intravenously administered free CMB [33].

In another study, piperine (PIP)-loaded PEG-PLGA NPs were formulated for the targeted delivery of adjuvant breast cancer therapy. Manendra Pachauri et al. reported that the PIP/PEG-PLGA NPs killed MCF-7 cells by apoptotic mechanism. When used in combination with PTX, the PIP-loaded NPs exhibited significant reduction in the PTX dose. Results obtained from the study led to the conclusion that the PIP/PEG-PLGA NPs were safe to be used as adjuvant cancer therapy against resistant breast tumours [34]. In 2019, Ankit Saneja et al. carried out a study where they formulated GEM and betulinic acid (BA) loaded PLGA-PEG NPs. Their aim was to improve chemotherapeutic efficacy. The NPs were formulated using double emulsion technique and had a resultant size of <200 nm. In vitro cytotoxicity tests were carried out on Panc1 cells and the results showed improved cancer cell toxicity as compared to free drug solution. In vivo anti-cancer activity was assessed on GEM/BA NPs in comparison to GEM/BA injection solution. The results obtained showed that the solid tumour treated with GEM/BA NPs had a mean volume of 195.5 mm^3^, while the tumours treated with GEM/BA injection solution had a mean volume of 213.5 mm^3^. The group concluded that the GEM/PLGA-PEG/BA NPs could potentially be used to co-deliver chemotherapeutics to enhance antitumor efficacy [35].

### 2.3. Polysaccharide-Based Biopolymers

Polysaccharide biopolymers consist of repeating simple sugar units joined by glycoside bonds. They are generally inexpensive and abundantly found in nature. They are stable, non-toxic and are excellent candidates for development of biocompatible anti-cancer drug delivery platforms. Biopolymers such as chitosan, alginates and hyaluronic acid have hydrophilic groups that form bio-adhesions with biological tissues, potentially increasing cellular drug retention which is beneficial in oral delivery of chemotherapeutics [36]. Additionally, they can be used to synthesise interpenetrated polymer networks with the desired physicochemical properties. All these properties make polysaccharide-based biopolymers excellent materials for the formulation of “smart” anti-cancer drug delivery platforms that can improve drug solubility, prolong drug release and enhance targeted delivery [36].

#### 2.3.1. Chitosan

Chitosan is produced from purified and N-deacetylated chitin. Chitosan production can be optimised to control the final product properties [37]. Chitosan is biocompatible, biodegradable, muco-adhesive and is an efflux pump inhibitor and it enhances drug cell permeation [38]. Its muco-adhesive and efflux inhibitor properties enhance anti-cancer drug cell permeation, increase intracellular concentration and thus help improve chemotherapeutic efficacy. Chitosan has been widely examined as a potential oral absorption enhancer due to its mucoadhesive properties and capacity to open tight junctions between epithelial cells, therefore enabling transportation of macromolecular drugs through “well-organised” epithelia. 

The –NH_2_ and –OH groups of the chitosan can be used as sites for molecule modification. Chitosan modification can either be physical or chemical. Chitosan derivatives offer modified properties for controlled drug release and can impact the pharmacokinetic profiles of the loaded anti-cancer drugs [39]. Chitosan can be used to coat other biopolymers to impart or enhance muco-adhesive properties.

Drug release from the chitosan NPs tends to be pH dependent [40]. Chitosan and its derivatives can be used in the formulation of ODPs for anti-cancer drugs to increase drug dissolution.

Chitosan has been used to deliver drugs with low water solubility and acid-labile anti-cancer drugs. For example, tamoxifen (TFN), which is sparingly water soluble and is therefore a good candidate for oral drug delivery. In 2015, S Barbieri et al. developed lecithin-chitosan NPs loaded with TFN. The TFN-loaded NPs showed muco-adhesive properties and increased drug permeation across the intestinal epithelium. Intestinal permeation of the TFN-loaded NPs was assessed in comparison to tamoxifen citrate in ringer solution (160 μg/mL). The amount of TFN absorbed and transported through the intestinal epithelia, from the tamoxifen citrate suspension, after 4 h was 0.76 nmol. On the other hand, the amount of TFN absorbed from the TFN-loaded NPs was reported to be 1.5 times higher than that of the tamoxifen citrate suspension. When the NPs were used, TFN passed through the intestinal tissue without being transformed by the CYP 450 enzyme. Hence, more of the drug was able to be found on the receptor site [41]. In another study, Feng et al. also developed a potential ODP for anti-cancer drugs. They prepared chitosan/carboxymethyl chitosan NPs, loaded with DOX. The DOX/carboxymethyl chitosan NPs showed enhanced drug intestinal absorption, throughout the small intestine. Sprague–Dawley rats were used to test bioavailability of orally administered DOX loaded NPs (10 mg/kg), orally administered DOX aqueous solution (10 mg/kg) and intravenously injected DOX solution (2 mg/kg). Blood samples were collected from the tails of rats at predetermined time intervals. No significant DOX was detected in the plasma after oral administration of the free DOX solution, demonstrating poor absorption of the free DOX. The group intravenously treated with DOX solution, resulted in a maximum plasma concentration (582.89 ng/mL) immediately after administration. The *C_max_* value of DOX after oral administration of DOX loaded NPs was 208.07 ± 20.17 ng/mL, which was nearly 4-folds, being 2.3-folds higher than that of free DOX solution. The results demonstrated improved intestinal absorption of DOX, when loaded onto the chitosan/carboxymethyl chitosan NPs, and hence improved oral bioavailability. Organs were excised from the rats, 24 h post-administration. The rats treated with oral DOX loaded NPs showed accumulation of drug in the liver (5.87 μg/g tissue), spleen (3.65 μg/g tissue) and lungs (2.58 μg/g tissue). While the rats treated with DOX solution (oral and intravenous), demonstrated DOX concentrated in the kidneys. These results indicated that DOX/carboxymethyl chitosan NPs could prolong systemic circulation and the retention time in the mentioned organs and can therefore be used to target tumours in the liver, spleen and lungs [42].

In 2017, Asad Khan et al. synthesised and characterised carboplatin (CPN)-loaded chitosan NPs for the treatment of breast cancer. The CPT loaded NPs had an average size of 277.25 ± 11.37 nm and a zeta potential of 31 ± 3.14 mV with low polydispersity index. Maximum drug encapsulation was obtained at 58.43%. The CPT chitosan NPs exhibited significant blood compatibility. When analysed in vitro, the NPs showed improved cytotoxicity effects against MCF-7 cell line. The group concluded that the CPT chitosan NPs could be used as a potential candidate for cancer treatment [43]. In a more recent study, pH-responsive chitosan-grafted-poly(methacrylic acid)/graphene oxide (CS-g-PMAA/GO) NPs were formulated as a potential chemotherapeutic delivery platform. The NPs were loaded with DOX and the experimental results obtained showed improved biological and physiochemical properties of the drug. At 100 μg/mL, the CS-g-PMAA/GO NPS had comparable therapeutic efficiency, with that of free DOX in solution for injection. Both treatments demonstrated a tumour survival rate of approximately 30%. The CS-g-PMAA/GO NPS had an added advantage of controlled drug release, favourable biodistribution and reduced drug side effects. The CS-g-PMAA/GO NPS demonstrated increased drug release in acidic media. It is well established that the tumour microenvironment has acidic conditions, and therefore it was concluded that the NPs may be applied as a potential chemotherapeutic delivery system, compared to free DOX in intravenous solution [44].

#### 2.3.2. Alginates

Sodium and potassium alginates have emerged as one of the most extensively explored biomaterials. Their unique physical properties permit for sustained release and targeted delivery of drugs, thus making them favourable BPs for use in oral drug delivery for cancer treatment [45]. This is because of their muco-adhesive properties, biocompatibility, cytocompatibility, sol-gel transition properties, and biodegradation and chemical versatility properties [46]. Alginates chemical versatility properties are beneficial in oral chemotherapeutics delivery, as they can easily be cross-linked and modified to enhance anti-cancer oral drug delivery. Mucoadhesive properties improve anti-cancer therapy absorption in the intestinal wall, therefore improving drug oral bioavailability [46].

Lim Vuanghao et al. published a report on their use of biocompatible disulphide cross-linked sodium alginate derivative NPs, for targeted oral delivery of PTX to treat colon cancer. They formulated self-assembled cysteamine based disulphide cross-linked sodium alginate NPs, aimed at improving the delivery of PTX to the colon cancer cells [47]. The drug-loaded NPs were reported to have exhibited 77.1% EE and a cumulative drug release of 45.1% in pH 6 medium with GSH. PTX release was based on the breakage of the disulphide linkages in the NPs. The reduction process was catalysed by GSH (reducing agent) in the colonic environment. The GSH is found in abundance in colon cancer cells as compared to normal cells; therefore, the condition could help in the high release of PTX from the NPs. At 0.8 μg/mL, the PTX-loaded NPs successfully destroyed colon cancer cells and demonstrated no toxicity towards normal colon cells. On the other hand, at 50 μg/mL; the PTX released from these NPs also destroyed normal cells. Cell internalization into the HT-29 cells was > 70%. This data therefore prompted them to conclude that the formulated PTX-loaded NPs, showed increased therapeutic efficacy as opposed to the free PTX in solution and may be considered as potential ODP for chemotherapeutics targeting colon cancer, with reduced toxic effects.

Another group developed DOX loaded pH sensitive alginate derivative NPs, targeted to the liver. The DOX loaded glycyrrhetinic acid modified alginate-alginate complex (GA-ALG/DOX-ALG) NPs were formulated by self-assembly method. The formulated NPs showed a pH sensitive release profile of DOX, where at pH 7.4, less than 10% of the doxorubicin had been released, whereas 58.7% of the drug was released at pH 4. Guo et al. carried out an in vivo biodistribution study in Kunming mice. DOX biodistribution from the formulated NPs was assessed in comparison to that of intravenous solution DOX-HCL. Significantly different biodistribution in heart, liver, spleen, lung, and kidney tissues was observed in DOX-HCl and GA-ALG/DOX-ALG NP groups, 1 h after administration. The DOX concentration in the liver of the GA-ALG/DOX-ALG NP group reached 27.6 μg/g, which was significantly higher than that of DOX-HCl (8.1 μg/g). The higher concentration of DOX in the liver was due to glycyrrhetinic acid, having liver-targeting properties [48]. Liver targeting led to reduced DOX accumulation in the heart and kidneys, which consequently would lead to reduced cardiotoxicity and nephrotoxicity, as compared to using intravenous DOX-HCL. DOX release from the NPs was still observed 72 h after administering to mice, while free DOX-HCl was not detectable 24 h post-injection. In vivo tumour growth inhibition was conducted, and results were obtained by histological analysis of H22 tumour cells, sixteen days after drug administration. The tumor growth inhibition was 78.91% after treatment with GA-ALG/DOX-ALG NPs compared to 51.93% in the DOX-HCl group.

In 2014, Ghislain Garrait et al. developed a novel drug delivery system of chitosan NPs entrapped in alginate microparticles. The aim was to develop an ODP that can protect the encapsulated drug from degradation in the gastric tract. In vitro drug release was studied in simulated gastric and intestinal fluids. Regardless of the presence or absence of enzymes, no breakdown of the alginate microparticles was observed. However, in simulated intestinal fluid, with or without pancreatin, the alginate was rapidly disintegrated. The alginate microparticles disappeared within 15 min, therefore releasing the chitosan NPs for loaded drug release. Experimental results obtained showed pH-responsive release of drug, as only 5% of loaded drug was released in the stomach, and at intestinal pH, the drug was completely released. The developed platform proved to be a suitable vehicle to protect anti-cancer drugs from gastric degradation, after oral administration [49].

#### 2.3.3. Hyaluronic Acid

Hyaluronic acid (HA) is a linear macromolecular mucopolysaccharide, composed of alternating glucuronic acid and N-acetylglucosamine units [50]. The hydroxyl, carboxyl and N-acetyl groups, expressed on the HA, make it a good candidate for chemical modification to create a compound with desired properties, such as sustained drug release and improved drug targeting. HA is biocompatible, biodegradable, has high viscoelasticity, is non-immunogenic and can recognize specific receptors overexpressed in tumour cells [51]. HA receptor CD44 is expressed at low levels in normal epithelial, hematopoietic and neuronal cells. The CD44 receptor is overexpressed in tumour cells, thus making HA a good targeting agent for cancer drug delivery. However, HA is easily degraded. Hence, a nitroxide-containing substance or a hyaluronidase inhibitor must be used in formulation to protect HA from rapid degradation in vivo [52].

The three functional groups on the main chain of HA can be modified, thus, different anti-cancer drugs can be conjugated to the HA, to formulate HA–drug conjugates. HA–drug conjugates are usually covalently bonded prodrugs and are not easily cracked in the blood but break through enzymolysis or hydrolysis after reaching the target site. HA–drug conjugates can improve drug solubility and are therefore a good candidate for use in oral delivery formulations of anti-cancer drugs with low aqueous solubility. HA also imparts change in drug biodistribution and increase in vivo half-life, as it leads to increased drug accumulation in the tumour sites due to enhanced osmotic retention and exerts better drug efficacy [53].

In 2015, Cai et al. made a novel delivery system by conjugating cisplatin (CIS) to HA. CIS is widely used for the treatment of most solid tumours, but its use is limited due to the serious side effects. The novel delivery system formulated by Cai et al. led to an increase in the concentration of platinum in the lymphatic vessels, reduced systemic toxicity and side effects, all while inhibiting early tumour metastasis of breast cancer [54]. At pH 7.4, it is expected from the release experiment that most CIS in the NPs will remain in the carrier for a considerable time period. Drug release will occur at the site of endocytosis where the pH is slightly lower, as protonation will trigger release of absorbed drug molecules. Compared to the free CIS in intravenous solution, the HA–CIS conjugate showed increased plasma drug concentration, improved tissue distribution and greatly reduced renal toxicity. In vitro drug tissue distribution from the HA–CIS NPs demonstrated 8.31-fold higher distribution as compared to that of free CIS in intravenous solution, after 4 h of incubation. These results clearly indicate that the drug-loaded NPs were internalised by the cells.

In 2019, Nilkamal Pramanik et al. formulated HA-modified graphene oxide (GO) and iron oxide NPs for targeted delivery of PTX and DOX. The resultant NPs exhibited cytotoxicity against breast cancer cell lines, BT-744 and MDA-MB-231. DOX and PTX were successfully loaded and the drug-loaded NPs significantly killed CD44-expressing MDA-MB-231 cells but did not kill the BT-474 cells that did not express CD44. Iron oxide was used in the formulation of the NPs to enable magnetic hyperthermia tumour cell targeting. In conclusion, the functionalized NPs demonstrated improved efficacy in killing tumour cells and thus can be used as a potential platform in cancer therapy [55]. The study was not conducted in comparison to any existing intravenous chemotherapeutic.

#### 2.3.4. Pullulan

Pullulan is a hydrophilic biopolymer. It is produced by bi-morphic fungi, *Aureobasidium pullulans*. Pullulan is non-immunogenic, non-toxic, and non-carcinogenic and it exhibits liver specificity. It is a linear and unbranched polymer, made up of repeating malto-triose units connected by α (1→6) glycosidic bonds. It has essential physiological activity due to the vast number of hydroxyl groups found on the main chain. Its hydrophilic properties make it of great importance in delivering poor water-soluble anti-cancer drugs. [56,57,58,59]. Pullulan is an excellent carrier for anti-cancer drugs targeting the brain, liver, lungs and spleen [60]. Although pullulan already has great potential for application into various medical applications, further chemical modification has proven to be advantageous in increasing its utility in the biomedical field. Substitution of the hydroxyl groups with the desired functional groups can be carried out via various chemical reactions [61].

In 2017, Junhui Sui et al. synthesised pH-responsive pullulan-DOX conjugate NPs encapsulating sorafenib (SNB) (P-Dox/S). The formulated NPs were developed as a synergistic delivery system for the treatment of murine breast carcinoma. Characterisation results showed a drug loading capacity of 65.34% (*w*/*w*). The NPs remained stable in physiological conditions. Cell toxicity of P-Dox/S was analysed in comparison to free DOX-HCL, in vitro against 4T1 cells. A stronger DOX signal was demonstrated in the nuclei of cells treated with P-Dox/S, after 0.5 h of incubation. The cell nucleus of the group treated with P-DOX/S began to fade and apoptosis after 6 h of incubation, with nucleus almost disappearing after 17 h. In vivo tumour accumulation and biodistribution of the NPs were tested in 4T1 tumour-bearing nude mice, in comparison to another group treated with free DOX-HCl. Compared with the free DOX-HCl treatment group, the fluorescent signal accumulation of NPs groups was stronger and lasted longer. This may have been due to liver-targeting properties of pullulan, which was the major component of the nanoparticle carrier. These results led to the conclusion that co-delivery of the chemotherapeutics outperformed the conventional delivery of single/free drugs. Combination therapy has vast potential for use in the treatment of tumours exhibiting drug resistance properties [62].

### 2.4. Biologics as ODPs for Anti-Cancer Drugs

Biological drug delivery platforms composed of living systems are a promising approach to deliver therapeutics. Biological systems have great potential to be used for the delivery of chemotherapeutics as they naturally operate with the central goal of controlled drug delivery and targeted delivery. Biologics such as RNA, DNA, exosomes, erythrocytes and leucocytes can be used as ODPs, as the anti-cancer drugs can be conjugated or encapsulated into the biologic platform [63]. However, oral delivery of biologics is challenging because the biological systems are readily degraded by proteases, nucleases and other enzymes in the gastric tract. Hence, to overcome this, the biologic platforms are usually conjugated with other biopolymers.

In this section, the use of RNA/DNA delivery platforms will be discussed. We briefly look into their current use as oral delivery platforms for chemotherapeutics and promising future prospective.

#### RNA/DNA Platforms for Chemotherapeutic Delivery

Research to date encompasses the conjugation of anti-cancer drugs with small interfering RNAs (siRNA), such as, PGP, XLAP, VEGF, BCL2 and MRP-1. siRNA have shown great potential for overcoming drug-resistant genes, as well as improving sensitivity of the resistant tumours to chemotherapeutics. It is noted that the siRNA must be protected from RNAse degradation and there is a need to prevent off-target side effects. Hence, the use of nanotechnology-based delivery [64]. siRNA refers to a group of synthesized duplex RNA, approximately 20–24 nucleotides in length, that can be recognized by the enzyme DICER, thus resulting in gene-specific cleavage by complementarily pairing with mRNA [65,66].

## 3. Nano-Enabled Biopolymer Systems for Specialised Oral Delivery of Anti-Cancer Drugs

In addition to the numerous engineered NP formulation platforms, nano-enabled systems are also used to develop chemotherapeutic delivery formulations. Liposomes, dendrimers, micelles, hydrogels, and metallic and carbon-based carriers can be used to increase chemotherapeutic efficacy, control drug release and improve cell targeting thus reducing cytotoxicity to normal cells [67]. Each carrier system has its own unique strengths. The following section concisely discusses each nano-enabled biopolymer system, chemical structure and use in chemotherapeutic delivery.

### 3.1. Dendrimers

**Figure 3:**
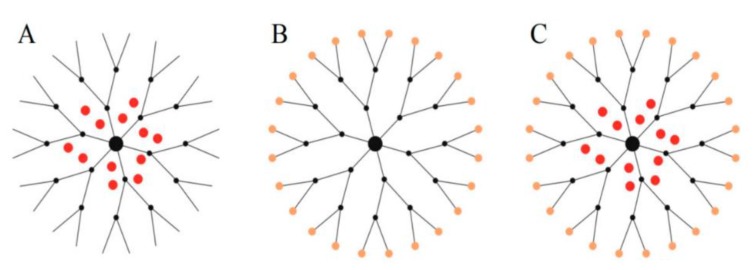
Schematic illustration of the three ways of complexation or conjugation of anti-cancer drugs with a dendrimer molecule. (**A**) Encapsulation of the chemotherapeutic into the internal cavity, (**B**) surface conjugation of chemotherapeutic onto the peripheral of the dendrimer molecule, and (**C**) same dendritic molecule can serve a carrier for both encapsulated and surface-conjugated anti-cancer drugs. Adapted and modified with permission from [71].

Dendrimers are branched polymeric molecules, which consist of uniform and well-defined shapes and sizes. Dendrimers basic structure consist of three main components—that is, the core, repetitive branching units and terminal groups [68]. The architecture of dendrimers can be controlled, and this makes them attractive molecules for use as drug delivery systems [69,70]. Hydrophobic anti-cancer drugs can be encapsulated in the dendrimers internal cavity or bound to the dendrimers surface groups through hydrophobic or electrostatic interactions as illustrated in Figure 3A–C.

There are three types of dendrimers, namely, poly-amidoamine (PAMAM), poly-propylene imine (PPI) and poly-l-lysine (PLL). They all exhibit good biocompatibility, water solubility, flexibility and biodegradability. Dendrimers can be conjugated with other polymers, for example, PEG. Conjugation with other polymers such as PEG is not only used to reduce the cytotoxic effects of dendrimers, but it can also increase plasma circulation time and tumour accumulation, through enhanced permeability and retention effect [72]. Dendrimers can also be modified by conjugation with tumour-specific antibodies [73,74]. These modifications are advantageous in cancer treatment, as they lead to reduced cell toxicity and improved cancer drug efficacy at lower doses.

The internal cavities of dendrimers, characteristic of their structure, allow for interaction with poorly water-soluble cancer drugs. Sanyamkamdhorn et al. investigated the intramolecular interaction of dendrimers with DOX and TFN [75,76]. Results showed the formation of hydrogen bonds between the drugs and the –NH_2_ in the PAMAM cavity. The hydrogen bonds, along with electrostatic interactions with the surface amino groups, enabled physical encapsulation of the drugs. Drug encapsulation led to sustained release of the drugs [75].

### 3.2. Nanoliposomes

Liposomes are widely used in drug delivery for systemic administration of drugs. They are microscopic unilamellar or multilamellar particles, made up of membrane-like lipid layers [77]. Their ability to encapsulate both hydrophobic and hydrophilic drugs makes them a favourable carrier for anti-cancer drugs. There has been a lot of interest in the use of liposomes as carriers for chemotherapeutics to increase therapeutic efficiency and reduce toxicity to the normal cells, and their small size allows for ease of cellular penetration. Liposomes are naturally occurring phospholipid based amphipathic nanocarriers. When introduced to an aqueous environment, phospholipids self-assemble to form a bilayer with the hydrophobic tails facing each other and the hydrophilic heads facing the aqueous environment. The core formed entraps water and thus water-soluble anti-cancer drugs [77].

**Figure 4:**
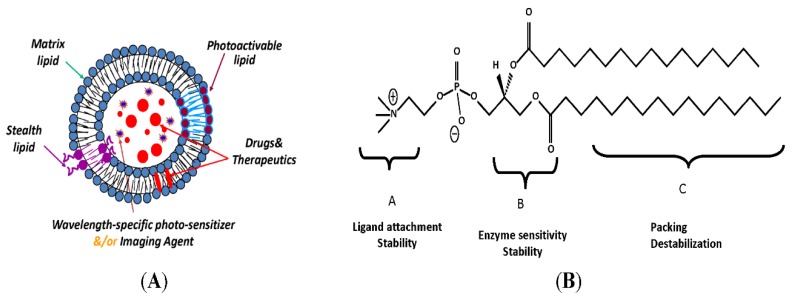
(**A**) Photo-sensitive liposomal assembly. Drugs (red), imaging agents and/or second photosensitizer (bright blue) are encapsulated in the liposomes. (**B**) Chemical structure shown as a prototype. The three lipid parts—that is, the head, glycerol backbone and fatty acyl chains—can be modified to generate photo-sensitive materials. Adapted and modified with permission from [81].

Active site targeting can be achieved by grafting the liposomes with monoclonal antibodies (mAbs), antibody fragments, peptides and glycoproteins [78]. Liposomes can be modified to be able to respond to both external and internal stimuli, such as pH changes, light (as shown in Figure 4A,B), microwaves, ultrasound and redox reactions [79]. They can also be radiolabelled to be able to determine their biodistribution and to diagnose the tumour. These kinds of liposomes that carry both therapeutic and imaging agents are called theranostic liposomes [80].

Joon Myong Song et al. performed an investigation on the liposomal co-delivery-based quantitative evaluation of chemosensitivity enhancement, in breast cancer stem cells [82]. They developed liposomes for the co-delivery of camptothecin (CTN) and glucose-regulated protein 78-siRNA/clusterin-siRNA. The drug–gene conjugate delivery was quantitatively assessed on the cancer stem cells. Compared to the free drug and gene deliveries, the co-delivery system showed that the cancer stem cells had increased transfection efficiency and chemosensitivity. They concluded that their data quantitatively demonstrated that using liposomes for the co-delivery of drugs and genes, offered substantial potential for synergistic anti-cancer therapy.

### 3.3. Nanohydrogels

A hydrogel is a swellable mesh of hydrophilic polymeric chains dispersed in water. Upon swelling, the hydrogels can release drugs previously loaded onto the mesh. Hydrogels have been and are currently being studied for use in oral delivery due to their polymeric chains which are able to closely interact with saliva glycoproteins [83]. The interaction with saliva glycoproteins causes a muco-adhesion phenomenon which leads to prolonged local retention of anti-cancer drugs, this characteristic may prove beneficial in the treatment of oral cancer. Due to the vast need to overcome disadvantages of intravenous administration for most chemotherapeutics, hydrogels have been investigated for the development of ODPs for drugs such as CIS, PTX, TFN and DOX [83].

### 3.4. Nanomicelles

Micelles are amphiphilic molecules which self-assemble when in contact with a solvent. If the solvent is hydrophilic, the polar parts of the copolymer will be attracted toward the solvent while the hydrophobic components orient away from the solvent. For this to happen, the amphiphilic molecules in solution must exceed critical micelle concentration [84,85]. A hydrophobic core is formed, and it facilitates the encapsulation of hydrophobic anti-cancer drugs, thus protecting the drugs from the external aqueous environment. On the contrary, when the amphiphilic molecules are exposed to a hydrophobic environment, a reverse micelle is formed.

A micellar system entrapped in a pH-responsive hydrogel was used by Wang L et al. to improve oral bioavailability of docetaxel (DTX). The polymeric micelles were formulated using poly-caprolactone (PCL) and PEG, to allow drug release in the intestinal lumen and subsequently absorption from the small intestine. The in vivo studies carried out showed a 10-fold increase in oral bioavailability of DTX and better tumour suppression against the 4T1 breast cancer cells used [86]. In another study, co-polymeric micelles composed of monomethylol PEG- PLGA, d-α-tocopheryl PEG 100 succinate and a stearic acid-grafted chitosan oligosaccharide loaded with DTX were formulated. The aim was to enhance oral bioavailability of DTX. The formulated DTX loaded micelles were analysed in vitro and the results obtained showed a 2.52-fold increase of drug oral bioavailability, compared to the free drug suspension [87].

### 3.5. Surface Modification for Targeted Delivery

**Figure 5:**
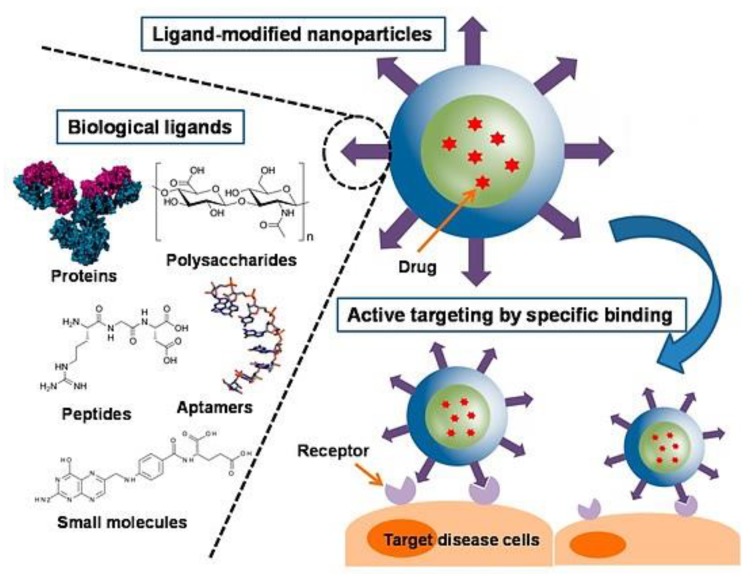
Schematic representation of a surface modified nanoparticle, highlighting examples of ligands used in surface modification and attachment to receptor site. Adapted and modified with permission from [89].

Nanotechnology delivery systems have gained momentum over the past few years due to their beneficial characteristics such as having a large surface volume ratio, drug protection from degradation, control of drug release and hence allowing for the reduction of administered drug doses. As favourable as the delivery systems are, they also have certain limitations. The chemotherapeutic agents carried by the nano-delivery systems are non-cell specific. The unwanted attack on healthy cells is the main reason for the failure of conventional nano-cancer treatment. Hence, nanotechnology delivery systems have had to further advance to develop novel drug delivery strategies that are able to overcome non-specificity issues, thus the use of targeting moieties as shown in Figure 5 [88].

Surface modification of the nano-delivery systems allows for active targeted delivery of the therapeutic agent into the tumours. There are several compounds that can be used for surface modification, for example, antibodies and ligands such as TF, folic acid (FA), lactoferrins, lectins and mannose derivatives.

FA the most commonly used compound for drug targeting applications, by conjugating it with anti-cancer drugs or nanocarriers. In one study, PAMAM dendrimers were amine-terminated and thus functionalised with FA and a fluorescein agent (isothiocyanate). This functionalised system acted as both a drug targeted system and a pH-responsive system for delivering DOX into tumour cells. The developed complexes displayed effective therapeutic efficacy and a high affinity for FA receptors, and thus were able to target the cancer cells and inhibit growth [90].

### 3.6. Stimuli-Responsive Nanoparticles

**Figure 6:**
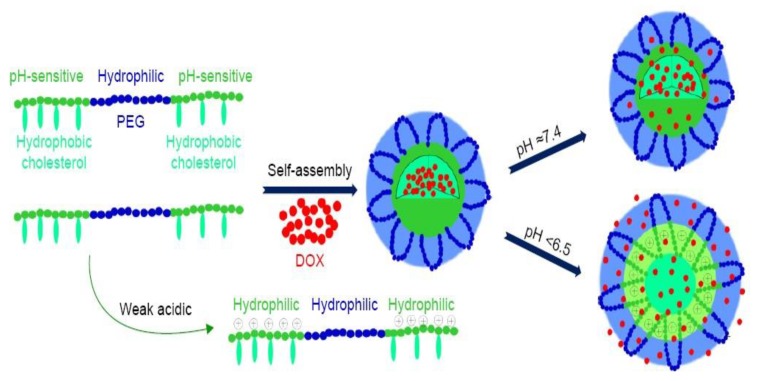
Schematic illustration of an example of self-assembled pH-responsive doxorubicin loaded micelles. The micelles were formulated using hydrophilic PEG and hydrophobic pH-sensitive PAE. The micelles will remain stable at normal sites (pH = 7.4), only to respond at the targeted site with the desired pH (pH < 6.5). Abbreviations: PEG—poly(ethylene glycol), PAE—poly(β-amino esters) g-cholesterol, and DOX—doxorubicin. Adapted and modified with permission from [92].

Apart from conjugating targeting compounds onto the delivery system, targeted chemotherapeutic delivery can additionally be accomplished by chemically modifying the biopolymer platform to respond to stimuli. Stimuli responsiveness is advantageous in cancer therapy as it ensures anti-cancer drug delivery at target site, hence sparing the healthy cells. Stimuli can be classified as either internal or external, for example, pH and temperature, activity of enzymes, light, irradiation and magnetic fields [91]. Chemical modification of the polymers leads to changes in the nanocarrier structure or chemistry, and thus triggers the release of the encapsulated drug in a biological environment, as shown in Figure 6. Chemically modified polymers respond to the slightest changes in the environment with notable transitions in the physiochemical properties, such as conformation, polarity, phase structure and chemical composition [91]. Change in physiochemical properties at the target site lead to enhanced diffusion and cellular uptake. Currently, bio-responsive drug delivery offers promising results for the delivery of nanoparticle chemotherapeutics.

## 4. Challenges with Targeted Delivery Challenges

Tumours show high intra-tumoral heterogeneity, resulting in heterogeneous expression of targets for drug delivery [93]. Targeted therapy is therefore not as simple as originally presumed. Only a subset of the tumour cells expressing the target is initially destroyed, while the tumour cells lacking the target survive and continue proliferating, resulting in a tumour lacking the initial expression target. Drug deliveries targeted at specific tumour cells tend to leave other tumour cells, most importantly, tumour stem cells. If the tumour stem cells, tumour-supportive cells and their supportive environment remain intact, it is therefore impossible to completely eradicate the whole tumour and prevent a relapse. Multi-targeting techniques therefore need to be considered in order to achieve complete tumour removal [93].

Another complex issue that arises when it comes to targeted drug delivery is the delivery of the entrapped drugs at their site of action. Current targeting systems simply aim to offload the drug at the site of action, working on the theory that, once the delivery system is bound to the target (e.g., membrane receptor), rapid internalisation occurs. Once internalisation has occurred, the drug is expected to be released and subsequently move to its site of action inside the cell, and not diffuse back into circulation [94]. However, the steps to deliver chemotherapeutics are more complex.

Even though targeting the tumour subsequently leads to internalisation at the target site, a wide range of drug delivery systems struggle to offload the drug because of failure to escape from the lysosomal compartments after internalisation [94,95]. Lysosomal sequestering of drug delivery systems which occurs following internalisation, is the main barrier preventing chemotherapeutics from reaching their target site. For example, less than 1% of administered PEGylated liposomal DOX (intravenous), reaches the target site i.e., the nucleus [96]. The rest of the internalised drug delivery system is found to be entrapped in the lysosomes.

Impaired release of chemotherapeutics may limit treatment efficacy. Therefore, alternative strategies to tackle these issues are needed. 

## 5. Regulation and Reality

Several polymer materials have already been approved for use as drug delivery materials by the Food and Drug Administration (FDA). However, the regulatory body is having a hard time keeping track with the rapidly increasing development of new polymer-based delivery systems. As of 2015, most of the FDA-approved delivery vehicles were either fully liposomal or PEGylated, with none of them addressing higher-level delivery complexities [97].

With regards to the development and approval of more novel strategies, more data are required from the scientific community to prove efficacy of such formulations. The FDA also needs to play its part with a greater sense of urgency in order to keep track of these developments. The burden of proof is assumed to mainly rest on academia as well as private and public sector research facilities, in order to identify the critical factors relating to safety and efficacy of the novel products and strategies [97].

The FDA generally classifies liposomes, dendrimers, micelles, nanoparticles and quantum dots as nanocarriers. From these, combination nanocarrier products have vastly gained interest over the years, leading to the development of co-encapsulation of synergistic cancer agents. The majority of the polymers of the formulations for cancer treatment, in clinical trials or having made their way into the market, are for intravenous use. There are currently several nanomedicines for cancer treatment on the market and in clinical trials. However, to date, a significant number of these formulations are for intravenous use. In addition, despite the FDA’s full inclusion of combination products on their regulatory evaluations, combination products typically have more hurdles to face before being accepted by the FDA.

The FDA has established regulatory guidance draft documents to provide recommendations for meeting the regulatory requirements. The FDA recommends that companies developing novel delivery systems carry out in vitro and in vivo studies and include details on the population to be studied, blood sampling times and analytes to be measured in those blood samples [97].

## 6. Conclusions and Future Perspectives

Interest in the application of biopolymers as oral delivery systems for chemotherapeutics is continuously increasing. This is due to polymers offering favourable characteristics, such as being biodegradable, biocompatible, non-toxic, hydrophilic and non-immunogenic. The use of biopolymer-based ODPs in cancer therapy allows for an increase in drug solubility, a reduction of dose, site-specific delivery and protection of the drug from the gastric environment. The drugs can be protected until they reach their site of absorption in the appropriate gastrointestinal segment for systemic circulation. Oral administration provides prolonged drug exposure, compared to intermittent intravenous infusion, which is an important factor for anti-cancer drugs. A drug with a short half-life can achieve greater systemic exposure by either prolonged infusion or by continuous oral dosing. Patients receiving chemotherapy prefer the oral route over the parental due to flexibility, convenience and the potential to reduce in-patient health care costs. Additional research comparing oral chemotherapeutics and parental chemotherapeutics are thus required as clinical trials. Hence, research further necessitates the use of biopolymers as ODPs for chemotherapeutics over a longer duration of evaluation. In addition, further studies are required to gather enough data in relation to toxicity of various polymers. Although many polymers show stability in vitro and ex vivo, these parameters may still need to be validated in vivo, using established diseased models. In the near future, ODPs have the potential to transform chemotherapeutic drug delivery, as they offer solutions to anti-cancer drug solubility issues, drug oral bioavailability and site specificity challenges.

## Figures and Tables

**Table 1:** Summary of biopolymers, their advantages and possible polymeric complexes.

Biopolymer	Advantages	Polymeric Complexes	Drug	Route	Animal Model	Tumour Type	Therapeutic Target	Entrapment Efficacy	Oral vs. Intravenous Drug Uptake	References
Gelatin	- Non-toxic - Biodegradable - Inexpensive - Can be cross-linked	- Redox-responsive gelatin nanoparticles - EFGR targeted gelatin nanoparticles	- Gemcitabine - Doxorubicin	- Oral	- SCID beige mice - Nude mice	- pancreatic adenocarcinoma - breast cancer	- Panc-1 cells - MCF-7 cells	- not reported - 82%	- *IG*_50_ 17.08 ± 2.32 μM vs. *IG*_50_ value of 8.39 ± 1.79 μM	[7,8,9,10,11,12,13,14,15,16,17,18]
Collagen	- Naturally occurring - Non-antigenic - Biodegradable	- Collagen nanoparticles	- Doxorubicin	- Oral	- In vitro study	- liver cancer	- Hep G2 cells	- not reported	- 48% vs. 22%	[19,20,21]
PGA	- Hydrophilic - Non-toxic	- PGA nanoparticles - PEG-b-(PGA)- b-poly(phenylalanine) nanomicelles	- Cisplatin - Doxorubicin	- Oral	- Female BALB/c mice	- ovarian cancer - lung cancer	- A2780 cells - NCI-H460 cells	- not reported	- *IG*_50_ 0.14 nM vs. *IG*_50_ 1.5 nM - not reported	[22,23,24,25,26,27]
PLGA	- Biodegradable - Biocompatible	- Magnetic PLGA nanoparticles - PLGA nanoparticles	- Paclitaxel - Cetuximab	- Oral	- Strain mice	- breast cancer - lymphoma tumour	- MCF-7 and U-87 glioma cells - DLS cells	- 82.9% and 87.3%	- 64% vs. 18%	[28,29,30,31,32,33,34,35]
Chitosan	- Biocompatible - Biodegradable - Mucoadhesive - Abundantly available	- Lecithin-chitosan nanoparticles - Chitosan nanoparticles	- Tamoxifen - Doxorubicin	- Oral	- Study carried out ex vivo - Sprague–Dawley rats	- general tumours	- not reported	- 60%	- not reported	[36,37,38,39,40,41,42,43,44]
Alginates	- Biocompatible - Sol-gel transition properties - Mucoadhesive - Non-toxic	- Disulphide cross-linked sodium alginate nanoparticles - -pH-responsive alginate nanoparticles	- Paclitaxel - Doxorubicin	- Oral	- Kunming mice	- colon cancer - liver cancer	- HT-29 and CRL 1790 cells - H22 cells	- 77.1%	- not reported - 1216.7 ng/mL vs. 657.7 ng/mL	[45,46,47,48,49]
Hyaluronic acid	- Biocompatible - High viscoelasticity - Non-immunogenic	- Hyaluronic acid nanoparticles	- Doxorubicin - Cisplatin - Paclitaxel	- Oral	- Female BALB/c mice	- ovarian cancer - breast cancer	- A2780 cells - CD44-expressing MDA-MB-231 cells	- 87 ± 5.6% - 68.76 ± 5.67% - 28.1 ± 7%	- not reported	[50,51,52,53,54,55]
Pullulan	- Hydrophilic - Non-carcinogenic - Non-toxic	- pH-responsive pullulan nanoparticles - Pullulan nanoparticles	- Doxorubicin	- Oral	- Nude mice	- breast cancer	- 4T1 cells	- not reported	- not reported	[54,55,56,57,58,59,60]
